# Comprehensive single-cell analysis reveals novel anergic antigen-presenting cell subtypes in human sepsis

**DOI:** 10.3389/fimmu.2023.1257572

**Published:** 2023-09-14

**Authors:** Tuo Zhang, Guodong Lian, Wei Fang, Lei Tian, Wenhao Ma, Jicheng Zhang, Zhaoli Meng, Hongna Yang, Chunting Wang, Chengguo Wei, Man Chen

**Affiliations:** ^1^ Department of Critical Care Medicine, Shandong Provincial Hospital, Cheeloo College of Medicine, Shandong University, Jinan, China; ^2^ Department of Gastrointestinal Surgery, Shandong Provincial Hospital, Shandong University, Jinan, Shandong, China; ^3^ Department of Gastrointestinal Surgery, Shandong Provincial Hospital Affiliated to Shandong First Medical University, Jinan, Shandong, China; ^4^ Department of Critical Care Medicine, Shandong Provincial Hospital affiliated to Shandong First Medical University, Jinan, China; ^5^ Division of Nephrology, Icahn School of Medicine at Mount Sinai, New York, NY, United States

**Keywords:** sepsis, antigen-presenting cells, dendritic cells, immunosuppression, monocytes

## Abstract

**Background:**

Sepsis is a life-threatening condition with high mortality. A few studies have emerged utilizing single-cell RNA sequencing (scRNA-seq) to analyze gene expression at the single-cell resolution in sepsis, but a comprehensive high-resolution analysis of blood antigen-presenting cells has not been conducted.

**Methods:**

All published human scRNA-seq data were downloaded from the single cell portal database. After manually curating the dataset, we extracted all antigen-presenting cells, including dendritic cells (DCs) and monocytes, for identification of cell subpopulations and their gene profiling and intercellular interactions between septic patients and healthy controls. Finally, we further validated the findings by performing deconvolution analysis on bulk RNA sequencing (RNA-seq) data and flow cytometry.

**Results:**

Within the traditional DC populations, we discovered novel anergic DC subtypes characterized by low major histocompatibility complex class II expression. Notably, these anergic DC subtypes showed a significant increase in septic patients. Additionally, we found that a previously reported immunosuppressive monocyte subtype, Mono1, exhibited a similar gene expression profile to these anergic DCs. The consistency of our findings was confirmed through validation using bulk RNA-seq and flow cytometry, ensuring accurate identification of cell subtypes and gene expression patterns.

**Conclusions:**

This study represents the first comprehensive single-cell analysis of antigen-presenting cells in human sepsis, revealing novel disease-associated anergic DC subtypes. These findings provide new insights into the cellular mechanisms of immune dysregulation in bacterial sepsis.

## Introduction

Sepsis is a life-threatening disease caused by a dysregulated systemic response to infection, and it is one of the leading causes of death in intensive care units (ICU) worldwide (1, 2). Recent estimates indicate that there are approximately 50 million cases of sepsis annually, with 11 million sepsis-related deaths reported in 2017, accounting for around 20% of global deaths (2). Despite decades of research, no effective targeted therapeutics against sepsis have been developed, likely due to the heterogeneity of this disease, which is likely attributable to diverse pathogen species, different infection sites, and individual immune responses (3–5). To address this challenge, several studies have demonstrated the need to further dissect the sepsis-induced systemic immune dysregulation at the cellular and molecular level (6–8).

Although recent single-cell RNA sequencing (scRNA-seq) studies have focused on various immune cell types such as monocytes, T cells, natural killer cells, myeloid-derived suppressor cells, platelets and erythroid precursor cells, dendritic cells (DCs) have received relatively little attention (9–13). Anergy, a state of immune unresponsiveness, is prevalent in septic patients, and low major histocompatibility complex class II (MHC II) expression has been identified as a surrogate marker of anergic monocytes and DCs. The expansion of anergic antigen-presenting cells (APCs) plays a crucial role in sepsis-related immunosuppression, which is considered a leading cause of secondary infections and even death in septic patients (14, 15).

DCs are traditionally classified into conventional dendritic cells (cDCs), including cDC1 and cDC2, and plasmacytoid dendritic cells (pDCs). cDCs effectively present specific antigens to CD4+ and CD8+ T cells, while pDCs produce type I interferons in response to viruses (16). Monocytes are subdivided into CD14+ and CD16+ monocytes based on the expression of CD14 and CD16 (FCGR3A). CD14+ monocytes exhibit greater phagocytic activity, whereas CD16+ monocytes express more MHC II related genes, indicating a higher antigen-presenting ability. However, recent studies utilizing scRNA-seq have revealed significant heterogeneity within these traditional cell types. For instance, Maier et al. demonstrated the existence of a DC cluster referred to as ‘mature DCs enriched in immunoregulatory molecules’ (mregDCs), which was associated with uptake of tumour antigens (17). Additionally, previous studies have divided the cDC2 population into two subtypes: DC3, characterized by high expression of monocyte-associated genes and shown to be expanded in several inflammatory conditions, including atopic dermatitis, psoriasis, systemic sclerosis, and systemic lupus erythematosus, and DC2, which exhibits gene expression profiling similar to traditional cDC2 (18–20). Moreover, Reyes et al. demonstrated the amplification of an anergic subset of monocytes (MS1), characterized by low expression of MHC II, in sepsis (9). These findings emphasize the necessity of using scRNA-seq to investigate the internal heterogeneity of traditional cell types and explore the relationship between cell subtypes and human disease pathogenesis. However, a single-cell resolution study specifically addressing the heterogeneity and dysfunction of APCs in human sepsis is still lacking.

Here, we conducted a comprehensive analysis of APCs in septic patients and identified three anergic DC subtypes that were significantly amplified in these patients. Furthermore, we observed that Mono1, previously reported as MS1 (9), exhibited expansion during the immunosuppressive stage and shared transcriptional regulatory similarities with anergic DCs. To validate our findings, we performed bulk RNA sequencing (RNA-seq) and flow cytometry on both septic patients and healthy controls. Our study provides valuable insights into the immune landscape of APCs in human sepsis and identifies new anergic APC subtypes. These findings have important implications for understanding and controlling immune dysfunction in sepsis.

## Material and methods

### Enrollment and sample collection

The study protocol was approved by the Human Biomedical Research Ethic Committee of Shandong Provincial Hospital. Written informed consent was obtained from the patients. Inclusion criteria for septic patients were based on the Third International Consensus Definitions of Sepsis and Septic Shock (Sepsis 3.0) and required standardized anti-infective therapy (21). Patients under 18 years old, pregnant or breastfeeding, those with chronic liver or kidney diseases, or those with incomplete medical records were excluded from the study.

For bulk RNA-Seq, peripheral blood samples were collected from 5 healthy controls and 20 septic patients who were admitted to the ICU of Shandong Provincial Hospital in China. The baseline characteristics of patients in the study are shown in **Table S1**. A volume of 2.5 ml of blood was collected from each participant into PAXgene Blood RNA tubes (BD Biosciences) to ensure the stability of intracellular RNA. Total RNA was isolated from PAXgene Blood RNA tubes using the PaxGene Blood miRNA kit (Qiagen). RNA sequencing libraries were prepared using the NEBNext UltraTM RNA library prep kit. The libraries were then analyzed on an Illumina NovaSeq 6000 platform.

For flow cytometry analysis, mononuclear cells were isolated from peripheral blood using density centrifugation. Each replicate included a total of 10 healthy controls and 20 septic patients. The experiment was performed in triplicate, resulting in a total of three replicates.

### Bulk RNA-seq data processing

TrimGalore was used to trim raw reads, and the trimmed reads were mapped to the hg19 genome using HISAT2, generating sam files that were then converted to bam files by SAMtools. HTSeq was used to calculate the read count of each gene, and R package edgeR was employed to identify differentially expressed genes (DEGs) using a cutoff of adjust.p < 0.05 and |log2FC| > 1 (22).

### scRNA-seq data processing

The scRNA-seq data were downloaded from the Broad Institute Single Cell Portal and analyzed using the R package Seurat (23). Cell quality control was applied based on four metrics: total unique molecular identifiers (UMI) counts, the number of genes detected, the expression ratio of hemoglobin genes and the expression ratio of mitochondrial genes. Cells were filtered out if they met any of the following criteria: (1) more than 25000 UMI counts; (2) more than 4000 detected genes or less than 500 detected genes; (3) more than 20% of mitochondrial genes; (4) more than 1% of hemoglobin genes. R package DoubletFinder was used to remove doublets on a per-sample basis, and then all ribosomal and mitochondrial genes were removed to avoid unexpected noise (24).

### Batch correction and cell subtypes annotation

To correct for batch effects in the dataset, the Harmony algorithm was used, and 3,000 variable genes were identified using the ‘SCTransform’ function (25). Principal component analysis (PCA) was performed using the ‘RunPCA’ function, and the PCA matrix was fed into the ‘RunHarmony’ function. Clustering and dimensionality reduction were then performed using the ‘FindClusters’ and ‘RunUMAP’ functions, respectively, based on the batch-corrected matrix.

Following the first round of clustering with a resolution of 0.8, 13 major cell types were identified, including B cells, plasma cells, NK cells, CD4+ T cells, CD8+ T cells, CD14+ monocytes, CD16+ monocytes, megakaryocytes, AXL+ SIGLEC6+ dendritic cells (AS-DCs), pDCs, cDC1s, cDC2s and cycling cells. Clusters expressing marker genes of more than one cell type were excluded and a total of 82273 cells expressing 22661 genes were retained for the downstream analysis. In the analysis for DCs and monocytes, each DC type and monocytes was extracted and underwent a second-round clustering with the same procedure as the first round to improve the annotation results.

### Analysis of DEGs from scRNA-seq data

Differential gene expression analysis was conducted using the ‘FindAllMarkers’ or ‘FindMarkers’ functions in R based on the ‘RNA’ assay, with a Wilcoxon signed-rank test used as the statistical method. A cutoff of adjusted P < 0.05 and |log2FC| > 0.25 was applied identify genes that were significantly differentially expressed.

### Module analysis of scRNA-seq data

For module analysis of scRNA-seq data, we utilized the R package high dimensional WGCNA (hdWGCNA), which is an extension of WGCNA (26). Specifically, we focused on cDC2 and monocytes and applied the analysis pipeline using the R packages Harmony and Seurat. The analysis pipeline included pooling cells within the same group and cell type to create metacells using the ‘MetacellsByGroups’ function, identifying an appropriate soft threshold using the ‘TestSoftPowers’ function, and selecting the module with the highest correlation to sepsis for further evaluation of its function using GO enrichment analysis. We also calculated the preservation of cDC2-related modules in pDC and cDC1 using the ‘ModulePreservation’ function and projected the cDC2-related modules and monocytes-related modules into other APC types using the ‘ProjectModules’ function.

### GO enrichment

GO enrichment analysis of DEGs was performed using R package clusterProfiler (27).

### Transcriptional factor analysis

To analyze transcription factor (TF) activity, we utilized the python single-cell regulatory network inference and clustering (PYSCENIC) tool on all single cells (28). Differentially expressed TFs were calculated using R package Limma (29). Only significant differentially expressed TFs with   adjusted p-value < 0.05 were involved in downstream analysis.

For prediction of differentially expressed TFs in bulk RNA-seq data between healthy controls and septic patients, we used the R package DoRothEA (30).

### Gene set score analysis

For bulk RNA-seq data, gene set score was calculated using the R package gene set variation analysis (GSVA), and in the case of scRNA-seq data, we calculated the gene set score using the ‘AddModuleScore’ function (31). Score changes between two groups were evaluated using the R package ggpubr based on the Wilcoxon signed-rank test or Student’s t test.

### Gene set enrichment analysis

To compare the three newly identified anergic DC subtypes with the previously reported DC3, we conducted pairwise comparisons using gene set enrichment analysis (GSEA). The GSEA signature list used in the analysis was obtained from the Supplementary Tables of Villani et al. (18). ‘DC3 > DC2’ gene set contained genes highly expressed in DC3 (characterized by low MHC II expression), while ‘DC2 > DC3’ gene set contained genes highly expressed in DC2 (similar to traditional cDC2).

### Heterogeneity analysis of septic patients based on monocytes gene expression profiling

To analyze heterogeneity in septic patients, we merged cells by calculating the sum of monocyte gene expression for each sample. Then, we corrected the gene expression of each sample using the ‘SCTransform’ function and selected the top 3000 genes for downstream analysis. For dimensionality reduction, we performed PCA and utilized the Louvain clustering method to cluster samples. We also applied reversed graph embedding to uncover the potential pseudostages that could link the clusters of patients (32).

### Pseudotime inference

Pseudotime analysis was performed on cDC1, cDC2 and pDC subtypes in sepsis using the R package Monocle3 (32–34). Top highly differential genes were plotted along the inferred developmental trajectories.

### Intercellular communication analysis

Intercellular communication analysis was conducted using the R package CellChat (35). The ‘CellChatDB.human’ database was used for analysis. The control and sepsis groups were analyzed separately and then merged for downstream analysis. We did not account for the effect of cell proportion, as the scRNA-seq data were enriched for DCs.

### Bulk RNA-seq data deconvolution

Bulk-data deconvolution was performed using CIBERSORTX (36). The reference signature matrix was constructed based on the count matrix in ‘RNA’ assay of scRNA-seq data.

### Module analysis of bulk RNA-seq data

A weighted co-expression network was constructed using the R package Weighted Correlation Network Analysis (WGCNA), followed by a selection of genes with the top 75% variance (37). The appropriate soft power β was selected using the ‘pickSoftThreshold’ function. The expression matrix is then converted to adjacency matrix to identify modules based on the topological overlap. A hierarchical clustering dendrogram was further built and the genes were clustered into different modules. The modules with the highest correlation to sepsis or control were selected for functional evaluation through gene ontology (GO) enrichment analysis.

### Flow cytometry

All antibodies used for flow cytometry were mouse anti-human mAbs. Anti-human CD45 (Cat No: 340910), CD56 (Cat No: 345811), CD123 (Cat No: 564195), HLA-DR (Cat No:756414) were purchased from BD Biosciences. Anti-human CD3 (Cat No: 317321), CD19 (Cat No: 302239), CD88 (Cat No: 344304), CD89 (Cat No: 354120), CD14 (Cat No: 325618), CD45RA (Cat No: 304142), CD1c (Cat No: 331520), CD141 (Cat No: 344112) were purchased from BioLegend.

Samples were run on LSRFortessa (BD Biosciences) flow cytometer. Initially, all DCs, including pDCs, cDC1s and cDC2s, were gated based on CD45^+^CD3-CD56^-^CD19^-^CD88^-^CD89^-^. Subsequently, pDCs were specifically identified by gating on CD123^+^CD45RA^+^. Following the exclusion of pDCs, cDC1s were identified based on the expression of CD141, while cDC2s were identified based on the expression of CD1c. Data were analyzed with FlowJo v.10.8.1.

### Statistical analysis

All analyses were performed using R software v4.2.1. The Student's t-test is used to assess the statistical differences between continuous variables that follow a normal distribution, while the Wilcoxon signed-rank test is used to assess the statistical differences between continuous variables that do not follow a normal distribution. The p values for differential gene expression analysis, GSEA, and GO analyses were adjusted using the Benjamini & Hochberg method. In **Table S1**, continuous variables were presented as mean ± standard deviation or median with interquartile range depending on their normality. Categorical variables were presented as counts (percentages). The level of significance was set at 0.05.

## Results

### Construction of single-cell atlas of septic patients

**Figure 1:**
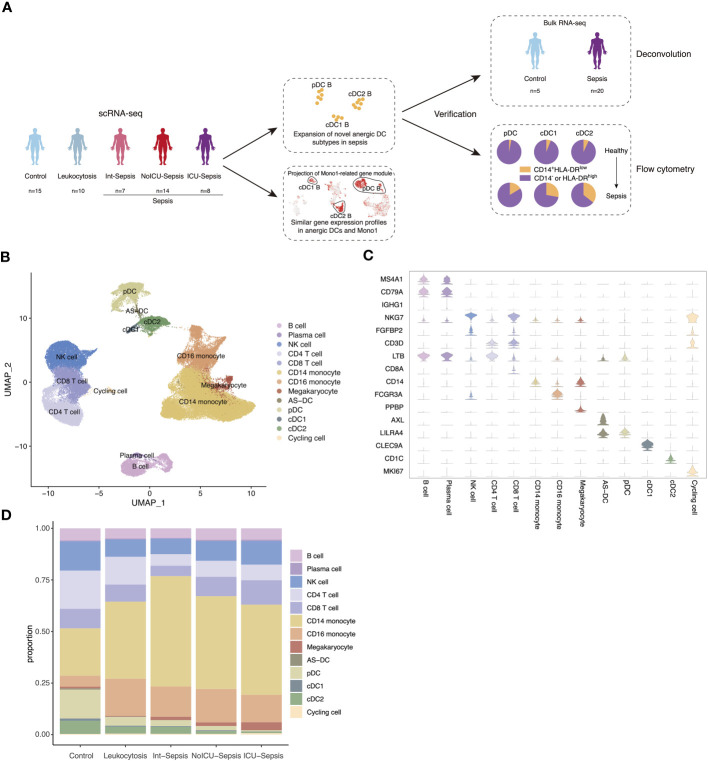
Single-cell atlas of septic patients. **(A)** Schematic diagram of study overview. Sample numbers indicated for each dataset. **(B)** Uniform manifold approximation and projection (UMAP) of cell types in the single-cell dataset of 82273 cells derived from septic patients and control participants. **(C)** Violin plots of canonical annotation marker genes (rows) for different cell types (columns). **(D)** Bar plot showing cell proportion in different groups.

We performed extensive literature mining at scientific search engines such as PubMed, Google Scholar, BASE, CORE to search related human sepsis scRNA-seq studies. Ultimately, we selected a study published in Nature Medicine with the largest sample size and DC enrichment for further analysis (9). The dataset was divided into 5 groups, including healthy control (n=15), leukocytosis (n=10, patients with blood WBC ≥ 12000 per mm^3^, but no organ dysfunction), Int-Sepsis (n=7, septic patients with mild or transient organ dysfunction), NoICU-Sepsis (n=14, septic patients in hospital wards or in emergency department), ICU-Sepsis (n=8, septic patients admitted to ICU). It is worth noting that all the groups except for leukocytosis and healthy control fulfilled the diagnostic criteria for sepsis (**Figure 1A**). After quality control and removal of doublets, we identified 13 cell types based on canonical annotation marker genes for immune cells used by many studies (**Figures 1B, C**). Our results showed that the proportion of monocytes increased gradually from the healthy control to the Int-Sepsis group, and then decreased as the disease progressed, while lymphocytes showed an opposite trend. Moreover, the proportion of DCs consistently decreased with disease progression (**Figure 1D**).

### Three anergic DC subtypes were identified in septic patients

**Figure 2:**
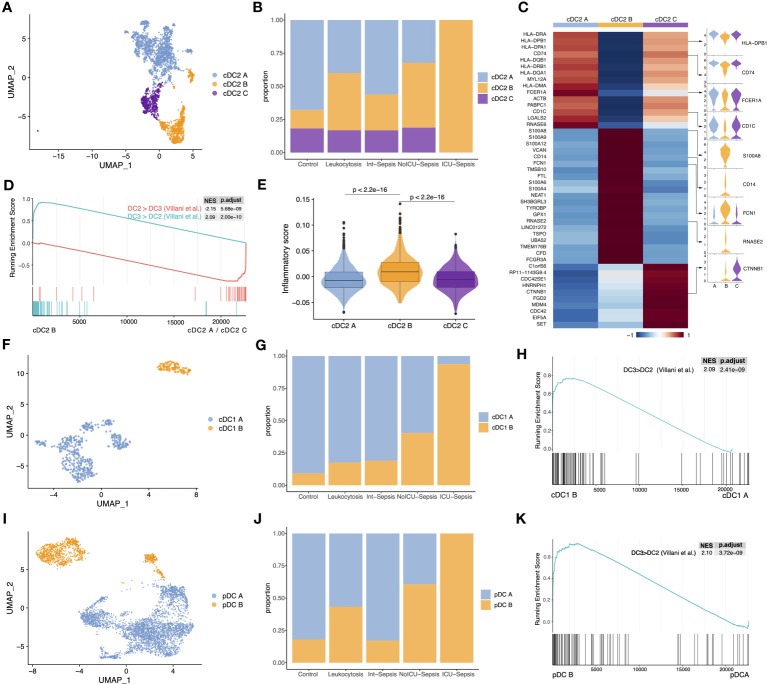
The proportion of three anergic DC subtypes significantly increased in sepsis. **(A)** UMAP presentation of three cDC2 subtypes. **(B)** Cell proportion of three cDC2 subtypes across different groups. **(C)** Heatmap of top marker genes across the three cDC2 subtypes (left). Violin plots for the expression of selected marker genes (right). **(D)** GSEA of pairwise comparisons of cDC2 B with cDC2 A and cDC2 C. ‘DC3 > DC2’ gene set contained genes highly expressed in DC3, while ‘DC2 > DC3’ gene set contained genes highly expressed in DC2. **(E)** Inflammatory score across three cDC2 subtypes. Significance was determined using Wilcoxon signed-rank test. **(F)** UMAP presentation of two cDC1 subtypes. **(G)** Cell proportion of two cDC1 subtypes across different groups. **(H)** GSEA of pairwise comparisons of cDC1 B with cDC1 A. **(I)** UMAP presentation of two pDC subtypes. **(J)** Cell proportion of two pDC subtypes across different groups. **(K)** GSEA of pairwise comparisons of pDC B with pDC A.

We next identified three cell subtypes, cDC2 A, cDC2 B and cDC2 C changed dynamically in sepsis disease progression in cDC2 population (**Figure 2A**). The populations of cDC2 A and cDC2 C gradually decreased, whereas the population of cDC2 B increased, particularly in the ICU-Sepsis group (**Figure 2B**). Additional analysis showed that cDC2 A and cDC2 C exhibited high gene expression levels of CD74 and HLA-DRA, which are associated with antigen presentation, the primary function of DCs. Conversely, cDC2 B was characterized by the expression of monocyte-associated genes such as S100A8, CD14, VCAN, and FCGR3A (**Figure 2C**). The marker genes of cDC2 B exhibited similarities to the previously reported DC3, whereas cDC2 A and cDC2 C were found to be more closely associated with DC2 (18). GSEA provided additional support for the idea that cDC2 B aligns with DC3 (**Figure 2D**) (18). Given that cDC2 B was previously identified as an inflammatory DC subtype (7), we calculated the inflammatory score of the three cDC2 subtypes and performed pathway enrichment analysis on the marker genes of cDC2 B. Interestingly, cDC2 B exhibited higher inflammatory score and enriched pathways related to the inflammatory response, such as ‘positive regulation of cytokine production’ and ‘positive regulation of inflammatory response’ (**Figures 2E** and **S1A**). Despite cDC2 B had a higher inflammatory score, we found that its highly expressed genes were monocyte-associated genes and it lost its primary function as a dedicated APC, specifically activating T cells via the MHC II pathway. Consistent with our findings, previous studies have demonstrated that cDC2 B has a reduced capacity to activate T cells in comparison to cDC2 A (7, 38, 39). Hence, we designated cDC2 B as anergic DCs, as previously described (15).

Next, we sub-clustered cDC1 population into two subtypes, cDC1 A and cDC1 B (**Figure 2F**). Surprisingly, cDC1 B, which shares similar marker genes with cDC2 B, was predominantly observed in the ICU-Sepsis group, suggesting the possible existence of an anergic subtype within the cDC1 population (**Figures 2G**, **S1B**). GSEA confirmed the similarity between cDC1 B and cDC2 B (**Figure 2H**). Moreover, analysis of inflammatory scores and pathway enrichment revealed that cDC1 B exhibited heightened inflammatory characteristics and enrichment in inflammatory pathways (**Figures S1C, D**).

Subsequently, we focused on pDC population to investigate whether the anergic subtype exists in all three traditional DC populations. We identified two clusters, named pDC A and pDC B (**Figure 2I**). Notably, pDC B, characterized by high expression of S100A8, S100A9, FCN1 and CD14, was the dominant subtype in the ICU-Sepsis group (**Figure 2J**). Further analysis using GSEA revealed a significant enrichment of the ‘DC3>DC2’ gene set in pDC B compared to pDC A (**Figure 2K**). Moreover, pDC B showed a higher inflammatory score and enrichment in inflammatory pathways (**Figures S1F, G**). These findings suggest that pDC B and cDC1 B represent two novel DC subtypes with gene expression profiling similar to the previously reported DC3, which we have designated as cDC2 B.

### Conserved transcriptomic profiles and regulatory mechanisms across cDC1 B, cDC2 B and pDC B

To further demonstrate the transcriptomic similarity of the three anergic DC subtypes, we performed hdWGCNA to identify co-expression gene modules in cDC2 and analyzed the preservation of these modules across other two DC populations (26). We identified a total of 15 modules in cDC2, of which five modules were found to be specific to cDC2 B, including the salmon, turquoise, magenta, red and greenyellow modules (**Figures S2A–C**). Of these five modules, only the red module was highly preserved in both pDC and cDC1 (**Figures S2D, E**). GO enrichment analysis revealed that this module was associated with inflammatory response and myeloid cell differentiation and activation (**Figure S2F**). We next projected the cDC2 gene modules onto pDC and cDC1 and found that the red module was also highly expressed in cDC1 B and pDC B, as well as in sepsis (**Figures S2G–J**). Furthermore, we performed trajectory and pseudotime analysis on all DC populations and found significant differences in gene expression between normal and anergic DC subtypes. The normal and anergic DCs were located at opposite ends of the pseudotime trajectory, with the expression of monocyte-related genes such as CD14, S100A8, S100A9, VCAN, and FCN1 increasing significantly along the pseudotime trajectory, while the expression of antigen-presentation-related genes such as CD74 and MHC II-related genes decreased significantly (**Figures S3A–F**). These findings further illustrate the consistency of the transcriptome among the three anergic DC subtypes and their close association with sepsis.

**Figure 3:**
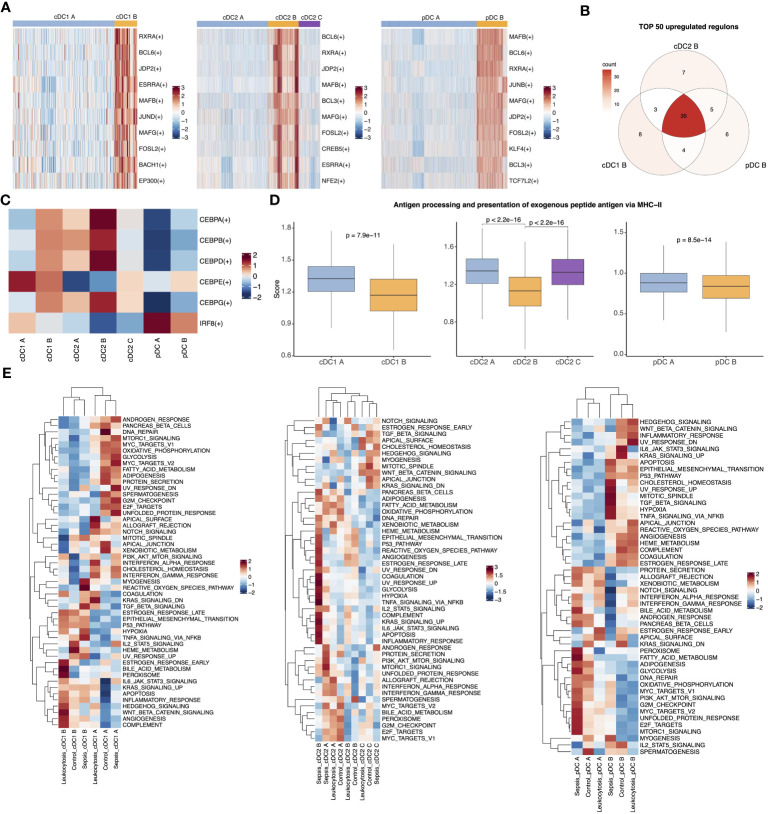
Three anergic DC subtypes shared similar upregulated TFs and enriched pathways. **(A)** Heatmap for top 10 upregulated TFs of pairwise comparisons of cDC1 B with cDC1 A (left), cDC2 B with cDC2 A (middle) and cDC2 C and pDC B with pDC A (right). **(B)** Venn plot showing the intersection of top 50 upregulated TFs in comparison of anergic DCs with their corresponding normal DC subtypes respectively. **(C)** Heatmap showed the activities of TF CEBP family and IRF8 across all DC subtypes. **(D)** Boxplot showed the antigen-presenting capacity via MHC II pathway. Significance was determined using Wilcoxon signed-rank test. **(E)** The heatmap of 50 MSigDB hallmark gene sets score across cDC1 B and cDC1 A (left), three cDC2 subtypes (middle) or pDC B and pDC A (right).

After demonstrating the transcriptome-level consistency of the three anergic DC subtypes, we then analyzed their TFs and pathway activity changes. Our analysis using PYSCENIC revealed that these anergic DCs had a high degree of similarity in the top upregulated TFs (**Figures 3A, B**) (28). Given the important role of the C/EBP family in inflammation-induced myelopoiesis and the high expression of monocyte-associated genes by anergic DCs, we evaluated the activity of C/EBP TFs (40). Notably, we observed a significant increase in the activity of almost all C/EBP family members in anergic DC subtypes. Furthermore, we discovered that cDC1 B and pDC B also exhibited low IRF8 activity, similar to the IRF8^low^ developmental pathway observed in cDC2 B (DC3), whereas cDC2 A/C (DC2) differentiated follows an IRF8^hi^ trajectory (41) (**Figure 3C**). These findings suggest that the three DC subtypes may share a similar developmental trajectory. We also examined antigen-presenting capacity changes between all DC subtypes and found that all three anergic subtypes had significantly lower ability to present external antigens via the MHC II pathway, indicating their anergic nature (**Figure 3D**). We then calculated the pathway activities based on 50 hallmark gene sets from MSigDB database to identify the pathway activity changes of all DC subtypes. Comparing the three anergic DCs with their corresponding normal subtypes, we observed similarities in pathway activity, including high activities in hallmark inflammatory pathways such as ‘INFLAMMATORY_RESPONSE’, ‘TNFA_SIGNALING_VIA_NFKB’, ‘TGF_BETA_SIGNALING’, ‘IL6_JAK_STAT3_SIGNALING’, ‘IL2_STAT5_SIGNALING’, and ‘COMPLEMENT’. Moreover, these anergic DC subtypes exhibited similarities in metabolic activity, including low oxidative phosphorylation, lipid metabolic activity and glycolytic activity (**Figure 3E**).

To investigate the distinct characteristics of anergic DCs in sepsis, we performed additional analysis to compare the phenotypic differences between septic patients and controls. The enrichment analysis showed that pathways related to antigen presentation were mainly enriched in the control and leukocytosis groups. In contrast, the sepsis group showed significant enrichment in inflammation-related signaling pathways, such as “positive regulation of cell activation,” “positive regulation of cytokine production,” and “NF-κB signaling” (**Figures S4A–F**). Similarly, a subsequent assessment of the antigen-presenting capacity of anergic DCs revealed that their ability to present antigens was enhanced in the leukocytosis group, but significantly reduced during sepsis (**Figure S4G**). Overall, our findings suggest that among the traditional DC types (cDC1, cDC2, pDC), there exist anergic subtypes with similar transcriptional regulation and enriched pathways. These subtypes may potentially contribute to the development of the observed immunosuppression in the context of sepsis.

### Gene expression similarities between immunosuppressive Mono1 and three anergic DC subtypes

**Figure 4:**
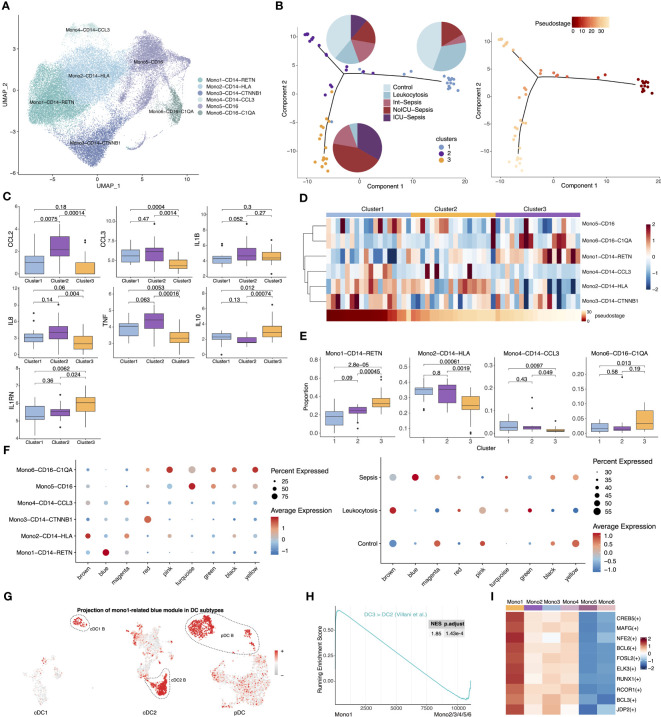
Comparison of gene expression profiles between immunesuppressive Mono1 and anergic DCs. **(A)** UMAP presentation of six monocyte subtypes. **(B)** Visualization of samples from septic patients colored by cluster (left) and pseudostage (right). Proportion of sample states for each cluster are shown in the left panel as pie charts. **(C)** Expression of pro-inflammatory and anti-inflammatory cytokines across three clusters. Significance was determined using Student’s t test. **(D)** Proportion of six monocyte subtypes across three clusters (top). Heatmap in the bottom showed ordered pseudostage for each sample. **(E)** Boxplots showing proportion of four monocyte subtypes (Mono1-CD14-RETN, Mono2-CD14-HLA, Mono4-CD14-CCL3, Mono6-CD16-C1QA) in each sample from the indicated clusters. Significance was determined using Student’s t test. **(F)** The expression level of the co-expression gene modules across six monocyte subtypes (left) and different groups (right). **(G)** The expression of Mono1-related blue gene module in cDC1, cDC2 and pDC subtypes. **(H)** GSEA of pairwise comparisons of Mono1 with other monocyte subtypes. **(I)** Top upregulated TFs of Mono1 in contrast to other five monocyte subtypes.

Monocytes have been extensively implicated in the development of sepsis, and the heterogeneity between patients is considered a major contributing factor to the ineffective immune modulatory interventions in sepsis (42). To investigate whether monocyte subtypes are associated with heterogeneity in septic patients, we examined the expression profiling of monocytes from patients. We isolated all monocytes and identified six subtypes, including Mono1-CD14-RETN, Mono2-CD14-HLA, Mono3-CD14-CTNNB1, Mono4-CD14-CCL3, Mono5-CD16, and Mono6-CD16-C1QA (**Figures 4A**, **S5A**). Notably, the proportion of Mono1, which shares similar marker genes with previously reported immunosuppressive MS1(**Table S2**, **Figure S5B**) (9), was significantly elevated in septic patients (**Figure S5C**). To investigate the covariation in gene expression patterns between patients, we merged the single-cell expression matrix into a sample-level matrix and used unsupervised analysis techniques, including dimensionality reduction, clustering, and pseudostage analysis, to identify three distinct clusters (cluster 1, cluster 2, and cluster 3) of samples that varied along a pseudostage. A three-stage model associated with different immune states was then developed (**Figure 4B**). Samples from cluster 2 exhibited high expression of pro-inflammatory cytokines, including CCL2, CCL3, IL1B, IL8 and TNF, while samples from cluster 3 showed high expression of immunosuppressive cytokines such as IL10 and IL1RN (**Figure 4C**). These findings suggest that peripheral inflammation in septic patients is highly heterogeneous. We then examined the composition of monocyte subtypes in the three clusters and observed a significant enrichment of different cell subtypes. Specifically, Mono1 and Mono6 were enriched in cluster 3, while the proportion of Mono2 and Mono4 decreased progressively in clusters 2 and 3 (**Figures 4D, E**). These results indicate that the differential abundance of certain monocyte subtypes may contribute to the observed differences in inflammation levels among patients.

Given the significant expansion of Mono1 in septic patients and its enrichment in immunosuppressive cluster3, Mono1 should be the one of the main contributors of sepsis. To further investigate the co-varying genes among Mono1, we employed hdWGCNA and identified nine modules (**Figure S5D**). Notably, we observed that the blue module exhibited high expression specifically in Mono1 and was exclusive to sepsis (**Figure 4F**). Surprisingly, GO enrichment analysis showed that the blue module was associated with myeloid cell activation and inflammatory response, which is highly similar to the cDC2 B-specific red module (**Figure S5E**). We therefore projected the Mono1-related blue module into all DCs and found that it was specifically expressed in anergic DC subtypes (**Figure 4G**). Furthermore, the cDC2 B-specific red module was highly expressed in Mono1 (**Figures S5F–G**). We then performed GSEA on monocyte subtypes and found that the ‘DC3 > DC2’ gene set was also enriched in Mono1. This suggests that Mono1 share a similar gene expression pattern with anergic DCs (**Figure 4H**). PYSCENIC analysis showed that the top 10 highly expressed TFs were also similar to those in anergic DCs (**Figure 4I**). In addition, Mono1 and anergic DCs shared similar transcriptional characteristics, such as low expression of MHC II-related genes. The onset of sepsis leads to the expansion of anergic subtypes within all APCs, which may be one of the main reasons for the development of immunosuppression in sepsis.

### Abnormal interactions of three anergic DC Subtypes and Mono1 with T cells

**Figure 5:**
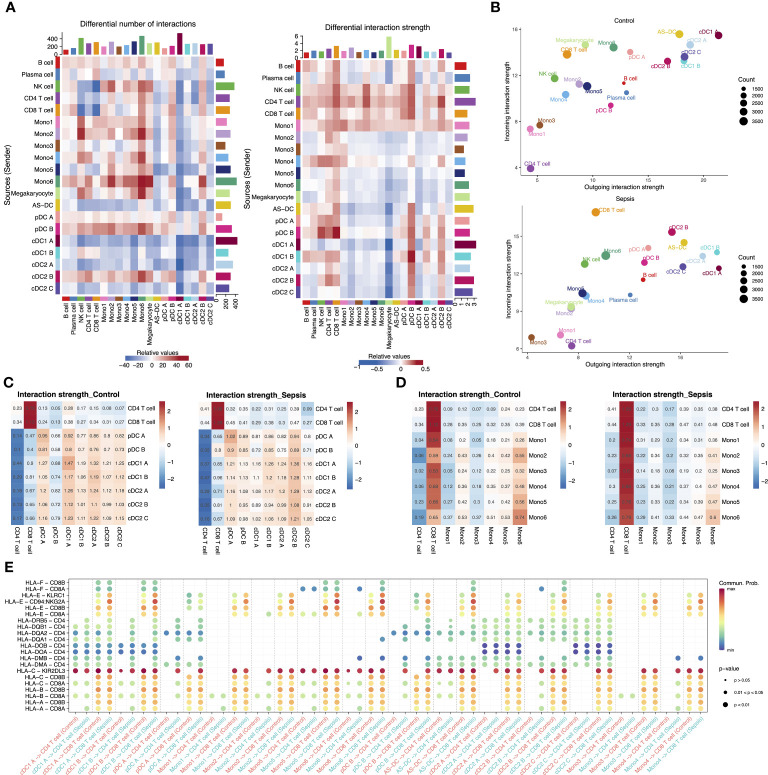
Intercellular communication in the peripheral blood of septic patients and healthy controls. **(A)** Heatmap showing the differential number of interactions among different cell types in sepsis versus healthy control (left). In the center, red stands for increased number of interactions, blue for decreased number of interactions. The bar plot above shows the sum of the changes in the number of incoming signals for each cell type. The right bar plot shows the sum of the changes in the number of outgoing signals for each cell type. The differential interactions strength among different cell types in sepsis versus healthy control (right). **(B)** Bubble plot showing the incoming and outgoing interaction strength for each cell type in healthy controls (top) and septic patients (bottom). The dot size represents the count of interactions. **(C)** Interaction strength of T cell subtypes and DC subtypes in control and sepsis. **(D)** Interaction strength of T cell subtypes and monocyte subtypes in control and sepsis. **(E)** Bubble plot showing the interaction possibility of all APC subtypes and T cells subtypes during control or sepsis through MHC I and MHC II pathway-related ligand-receptor pairs.

Based on our observation of reduced expression of the MHC II genes in APCs and their specific role in interactions with T cells, we hypothesized that sepsis may lead to a generalized dysfunction in APC-T cell communication. We then utilized CellChat to analyze communication patterns among different cell subtypes separately for each condition (35). We observed that CD4 T cells and CD8 T cells exhibited a significant increase in the strength of interactions in septic patients. Specifically, CD4 T cells showed a greater increase in outgoing signals than CD8 T cells, while CD8 T cells had a higher increase in incoming signals. Interestingly, normal DC subtypes (cDC1 A, cDC2 A, cDC2 C and pDC A) showed no significant enhancement in outgoing signals or even a decrease, while anergic DC subtypes (cDC1 B, cDC2 B and pDC B) exhibited significantly enhanced interactions with T cells (**Figure 5A**). Moreover, DCs were found to be the primary receivers and senders of signals regardless of the condition (**Figure 5B**). We then focused on the interactions between all APC subtypes and T cells. The strength of the interaction between these anergic DCs and T cells was weak in healthy controls, but significantly enhanced in septic patients, even exceeding that of the other DCs (**Figure 5C**). Additionally, the interaction between Mono1 and T cells was also significantly enhanced in septic patients (**Figure 5D**). Although the anergic DCs are less likely to act via ligand-receptor pairs in the MHC I and MHC II pathway compared to other DC subtypes, they showed enhanced communication probability to use these pathways in sepsis (**Figure 5E**).

### Validation of anergic DC expansion in sepsis through deconvolution analysis of bulk RNA-seq data and flow cytometry

**Figure 6:**
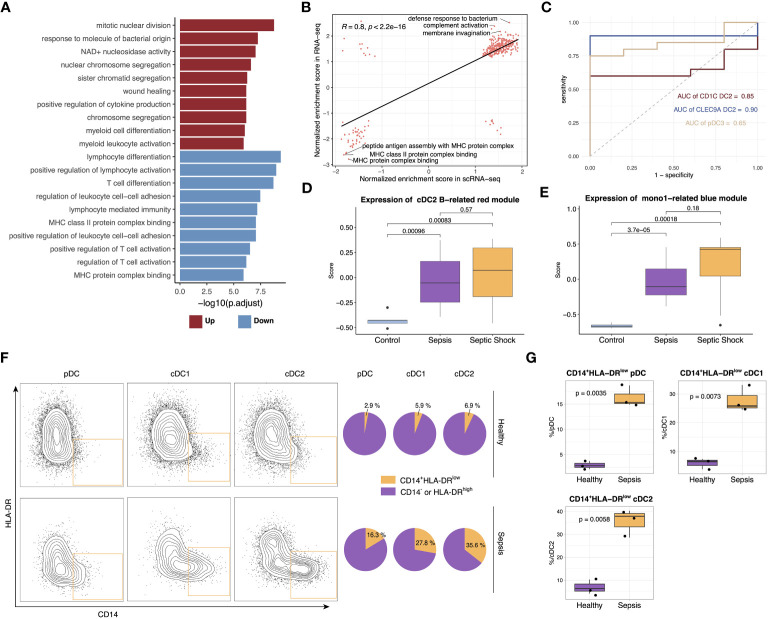
Verification of scRNA-seq findings with bulk RNA-seq and flow cytometry. **(A)** Top upregulated and downregulated GO terms in septic patients versus healthy controls. **(B)** Scatterplots showing correlation between pathway normalized enrichment score in scRNA-seq data and RNA-seq data. The pathway normalized enrichment score was calculated using GSEA. Pathways with p <0.05 were included in the analysis. Significance of the correlations (Pearson r) were calculated with a two-sided permutation test. **(C)** ROC curves for the diagnostic effectiveness of proportion of cDC1 B, cDC2 B and pDC B in all DCs in the bulk RNA-seq data. **(D)** Boxplot showing the expression of genes from cDC2 B-related red module in different groups. Significance was determined using Student’s t test. **(E)** Boxplot showing the expression of genes from Mono1-related blue module in different groups. Significance was determined using Student’s t test. **(F)** The frequency of CD14^+^HLA-DR^low^ DC subsets in the sepsis patients and healthy control. **(G)** Boxplot showing the difference in the proportion of CD14^+^HLA-DR^low^ pDC, cDC1 and cDC2 between sepsis patients and healthy controls. Significance was determined using Student’s t test.

To confirm the findings from the single-cell dataset, bulk RNA-seq analysis was conducted on 20 septic patients and 5 healthy controls. After quality control based on PCA and heatmap, we performed differential expression and GO enrichment analysis. The upregulated genes were chiefly enriched in cytokine release and myeloid activation, while the downregulated genes were mostly enriched in T cell activation and MHC II pathway, which is in alignment with the changes in cell proportions demonstrated in the scRNA-seq dataset (**Figure 6A**, **Figures S6A–C**). Furthermore, correlation analysis revealed a significant positive correlation between the pathway enrichment scores of the scRNA-seq dataset and the bulk RNA-seq dataset (**Figure 6B**). To assess the potential diagnostic value of these anergic DC subtypes in sepsis, deconvolution analysis was carried out. The analysis indicated higher proportions of the three anergic DC subtypes in septic patients compared to healthy controls. Additionally, the proportions of these anergic DC subtypes inferred by CIBERSORTX for each subject individually can be used as a classifier of sepsis (area under the curve (AUC), cDC1 B = 0.90, cDC2 B = 0.85, pDC B = 0.65) (**Figure 6C**). The expression level of the anergic DC-related red co-expression module and Mono1-related blue module in the bulk RNA-seq data was also evaluated. Consistent with cell proportions, the expression of both modules was significantly increased in septic patients, and further augmented when the patients presented with shock (**Figures 6D, E**). We next conducted WGCNA on bulk RNA-seq data to identify modules specific to healthy controls and septic patients (37). The analysis identified 13 distinct modules, of which three (purple, magenta, and tan) were specific to sepsis, and two (blue and turquoise) were highly expressed in healthy individuals. The purple module was strongly associated with exocytosis, myeloid cell differentiation, and endoplasmic reticulum stress, and exhibited significantly higher expression levels in sepsis patients. Additionally, its expression was observed to further increase in the presence of shock. The magenta and tan modules were linked to cell division and humoral immunity, respectively. Their expression levels were enhanced in septic patients but did not appear to be further increased at the onset of shock, and in fact, their expression levels even seemed to be reduced. The expression of the blue module showed a clear decrease with increasing disease, although this module was not enriched in any specific pathway. Finally, the turquoise module, related to antigen presentation and lymphocyte differentiation, displayed a consistent decrease in expression with increasing disease, in line with the results obtained from the differential analysis (**Figures S6F, G**). Given that the purple module demonstrated the strongest correlation with sepsis and its expression was further enhanced with disease progression, the expression of hub genes within this module was evaluated in different APC subtypes. Notably, these hub genes were significantly overexpressed in all three anergic DCs, as well as in Mono1, further supporting the relevance of these cells to sepsis (**Figure S6H**).

Following the insights gained from the single-cell analysis, we proceeded to perform flow cytometry analysis on cohort comprising both healthy individuals and septic patients. The detailed gating strategy steps employed in the analysis are provided in the Methods section. Our findings unveiled a notable elevation in the relative proportion of CD14^+^HLA-DR^low^ DCs specifically within pDCs, cDC1s and cDC2s in septic patients, thereby providing additional validation to our initial observations (**Figures 6F, G**).

## Discussion

Sepsis is a complex and life-threatening disease, and the lack of understanding of its pathological mechanisms at the single-cell level has hindered progress in this area. scRNA-seq provides an ideal tool for studying the mechanism of sepsis at single-cell resolusion. Several previous scRNA-seq studies on sepsis have been published, but none of these studies spotlighted APCs in human sepsis (9–13, 43). We therefore extracted DCs and monocytes from previously published scRNA-seq data to study the dysfunction of these cells during sepsis and their internal heterogeneity (9).

Through integrated analysis of scRNA-seq and bulk RNA-seq data from septic patients and healthy controls, we precisely delineated all APC subtypes and identified new anergic DC subtypes specifically amplified in sepsis.

Despite the clear decrease in the number of DCs during sepsis (14, 44), we observed a considerable increase in anergic cDC2 B within the DC population, which was characterized by low expression of MHC II. While anergic DCs are enriched in inflammatory pathways, previous studies in mice and humans have demonstrated that cDC2 B exhibit a reduced capacity to activate T cells compared to cDC2 A (7, 38, 39). Additionally, cDC2 B has been shown to have weaker migratory abilities (39). These findings provide further support for the anergic phenotype of cDC2 B cells. In a consistent manner, Villani et al. discovered two subtypes, namely DC2 and DC3, within the conventional cDC2 subset. Notably, our cDC2 B corresponds to DC3, whereas cDC2 A/C align with DC2 (18). The amplification of cDC2 B is not limited to sepsis, as it has also been observed in various other inflammatory diseases such as systemic lupus erythematosus, atopic dermatitis, and psoriasis (19, 20). Additionally, our analysis showed the presence of anergic subtypes within cDC1 and pDC, which displayed similar gene expression profiles to cDC2 B cells. All three anergic DC subtypes exhibited high expression of CD14. However, Villani et al. strictly excluded CD14+ cells from their DC sorting procedure, potentially resulting in the capture of only a small fraction of these dysfunctional DCs (9). Moreover, anergic cells within pDCs and cDC1s showed relatively less heterogeneity compared to regular DCs. Identification of these cells might require re-clustering analysis tailored to each DC population.

To further confirm the conservation of transcript levels among the three anergic DCs, we performed hdWGCNA on cDC2 and calculated the conservation of its co-expression gene modules in cDC1 and pDC. Our results indicated that the cDC2 B-related red module was highly conserved within all three traditional DC populations and was highly expressed in cDC1 B as well as pDC2 B. Using PYSCENIC analysis, we found that the three anergic DC subtypes also shared similarities in terms of transcriptional regulation. The development trajectory of DCs is greatly influenced by the activity of IRF8, a TF crucial for the normal development of cDC1s and pDCs in mice (45–47). IRF8, along with other TFs such as CEBPA and PU.1, helps balance the fate of neutrophils, monocytes, and DCs at various stages of development (48–51). Previous studies had shown that cDC2 B developed through an IRF8^low^ pathway, while cDC2 A/C followed an IRF8^hi^ pathway (41). In line with this finding, our study revealed that IRF8 activity was significantly decreased in cDC1 B, cDC2 B and pDC B. Additionally, we observed enhanced CEBP family activity in all three anergic DC subtypes. This suggests that the expansion of anergic DCs may be associated with altered myelopoiesis in sepsis, a phenomenon that has been reported in mice and simulated *in vitro* (52). While cDC2 B and cDC2 A/C were considered distinct DC subtypes with different developmental, transcriptomic, phenotypic, and functional characteristics, it remained unclear whether pDC B and cDC1 B were unique DC subtypes (41, 53).

Monocytes have been extensively implicated in the pathogenesis of sepsis (42). By analyzing the expression profiles of patient monocytes, we constructed a three-stage model that revealed distinct patient clusters characterized by varying levels of inflammation and immunosuppression. This emphasizes the critical need for personalized treatment strategies in sepsis, as the optimal therapeutic approach may depend on whether the patient is in a hyperinflammatory or immunosuppressive state. Furthermore, we observed the expansion of Mono1, which was characterized by low expression of MHC II, within the immunosuppressed cluster 3. These findings suggest a potential pivotal role of Mono1 in the development of immune paralysis during sepsis. Consistently, previous studies have consistently demonstrated the marked downregulation of monocyte HLA-DR expression in sepsis, which is closely linked to patient prognosis (54). Moreover, the observed similarities in transcriptome profiling between Mono1 and anergic DCs imply the existence of shared regulatory mechanisms governing the differentiation of monocytes and DCs in the context of sepsis. Further investigation is warranted to unravel the underlying intricacies of this phenomenon.

Despite improved survival rates during the hyper-inflammatory stage, sepsis continues to have a high mortality in ICU patients due to the development of prolonged immune suppression (55, 56). APCs play an important role in the development of the immunosuppressed state in sepsis. Previous studies have demonstrated a significant impairment in the ability of APCs to present antigens through the MHC II pathway in sepsis (42, 54). Our findings reveal that the decreased antigen-presenting ability of APCs can be attributed to the amplification of specific anergic subtypes. Previous studies have reported a decrease in the absolute number of DCs during sepsis (14, 44), indicating a reduction in the absolute count of anergic DCs. Nevertheless, the discovery of anergic DC subtypes still carries significant implications for sepsis treatment. While previous research on DCs has primarily concentrated on enhancing DC expansion (57, 58), improving DC survival and modifying DC function (59–62), the identification of anergic DC subtypes underscores the need to investigate the influence of the transition of DCs to anergic phenotypes on the progression of sepsis.

This study has some limitations. Firstly, further investigation is needed to explore the relationship between the absolute count of anergic DCs and the prognosis of septic patients. Furthermore, the underlying regulatory mechanisms governing the behavior of the three anergic DC subtypes and Mono1 during sepsis require further investigation. Additionally, it is yet to be determined whether the development of pDC B and cDC1 B follows a similar IRF8^low^ pathway as observed in cDC2 B, necessitating further research in this area.

## Conclusions

In conclusion, our study identified novel anergic APC subtypes in the pathogenesis of sepsis with integrative informatics analysis of scRNA-seq and following verification by bulk RNA-seq and flow cytometry. By revealing the heterogeneity and functional defects of APCs in sepsis, our work emphasizes the importance of understanding the cellular and molecular mechanisms of immune suppression. Moreover, the identification of these anergic APC subtypes highlights their potential as therapeutic targets for the treatment of sepsis. Overall, this study lays the groundwork for future investigations aimed at improving the clinical management of this devastating disease.

## Data availability statement

The datasets presented in this study can be found in online repositories. The names of the repository/repositories and accession number(s) can be found below: HRA004458 (GSA).

## Ethics statement

The studies involving humans were approved by Human Biomedical Research Ethic Committee of Shandong Provincial Hospital. The studies were conducted in accordance with the local legislation and institutional requirements. The participants provided their written informed consent to participate in this study.

## Author contributions

TZ: Data curation, Methodology, Software, Visualization, Writing – original draft. GL: Supervision, Validation, Writing – review & editing. WF: Supervision, Writing – review & editing. LT: Data curation, Writing – review & editing. WM: Data curation, Writing – review & editing. JZ: Writing – review & editing. ZM: Supervision, Writing – review & editing. HY: Supervision, Writing – review & editing. CTW: Supervision, Writing – review & editing. CGW: Supervision, Writing – review & editing. MC: Funding acquisition, Supervision, Writing – review & editing.
