# Supplementary material for: Comprehensive single-cell analysis reveals novel anergic antigen-presenting cell subtypes in human sepsis
Source: Front Immunol. 2023 Sep 14;14:1257572. doi: 10.3389/fimmu.2023.1257572 (PMC10538568; doi:10.3389/fimmu.2023.1257572)
Supplement: Supplementary file 1 [file DataSheet_1.pdf]

SUPPLEMENTARY FIGURES

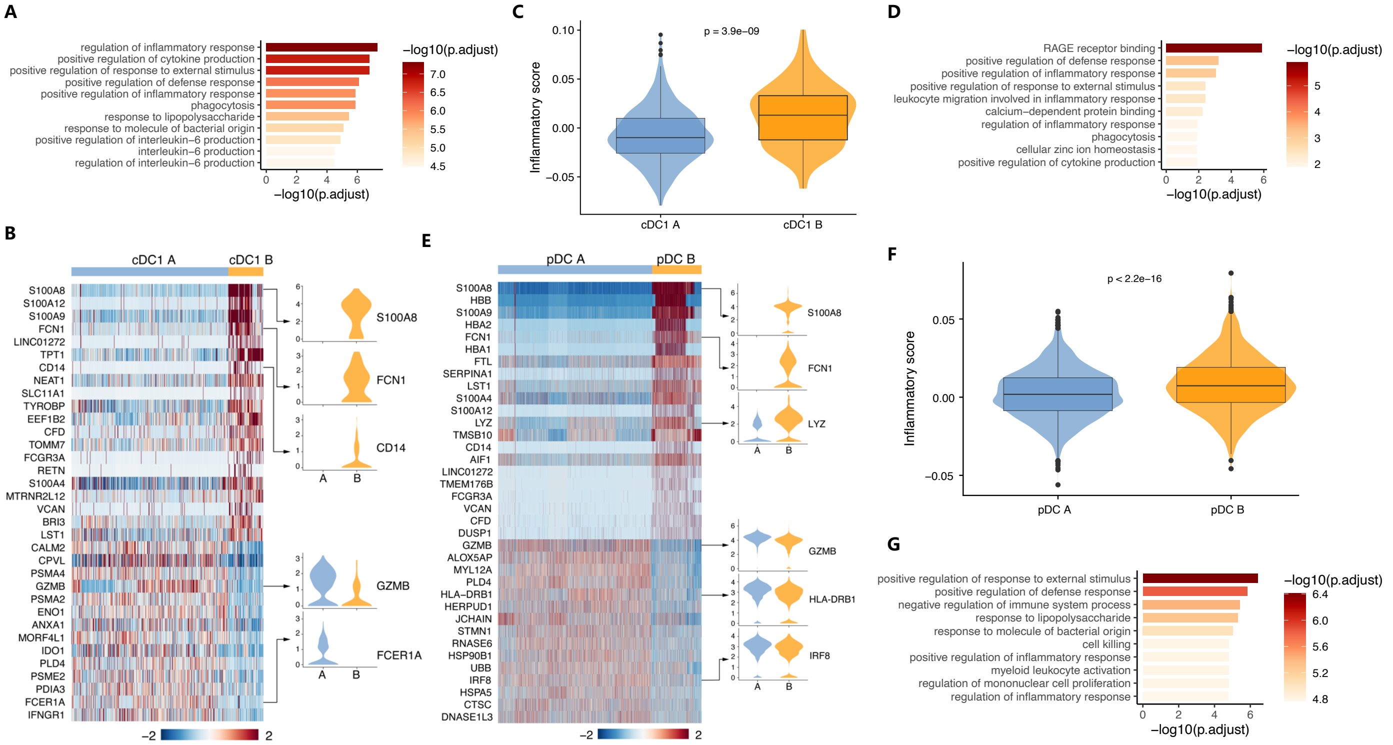

Figure S1. Transcriptome characteristics of three anergic DC subtypes. **(A)** Upregulated GO terms of cDC2 B versus cDC2 A and cDC2 C. **(B)** Top marker genes of the two cDC1 subtypes. Heatmap of the expression level of all marker genes across each cell (left). Violin plots showed the expression of selected marker genes across each cDC1 subtype (right). **(C)** Inflammatory score between two cDC1 subtypes. Significance was determined using Wilcoxon signed-rank test. **(D)** Upregulated GO terms of cDC1 B versus cDC1 A. **(E)** Top marker genes of the two pDC subtypes. Heatmap for the expression level of all marker genes across each cell (left). Violin plots for the expression of selected marker genes across each pDC subtype (right). **(F)** Inflammatory score between two pDC subtypes. Significance was determined using Wilcoxon signed-rank test. **(G)** Upregulated GO terms of pDC B versus pDC A.

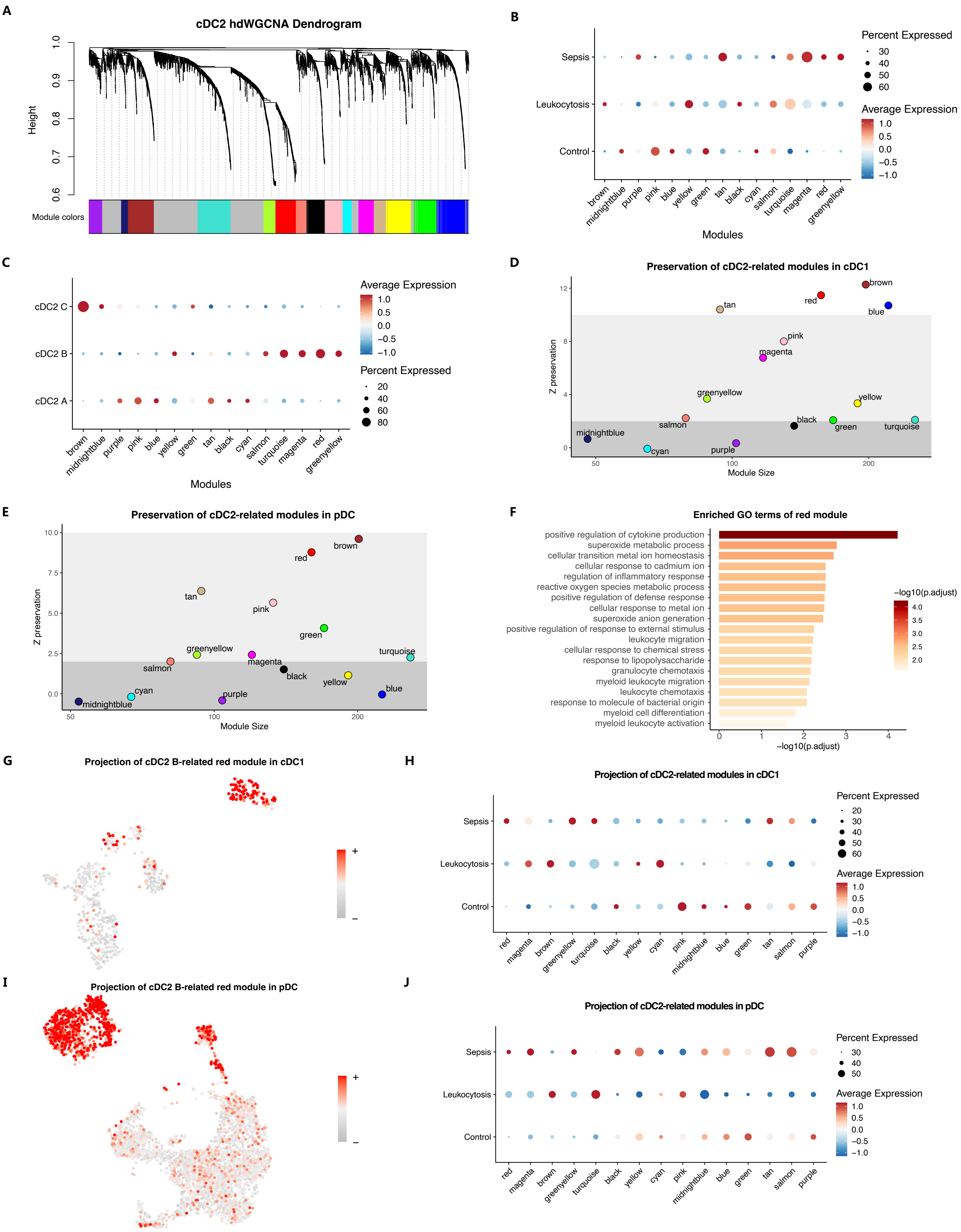

Figure S2. hdWGCNA on DC subtypes revealed anergic DC specific gene module. **(A)** The cluster dendrogram of co-expression genes of cDC2. **(B)** The expression level of each cDC2 gene module across different groups. **(C)** The expression level of each cDC2 gene module across three different subtypes. **(D)**

Module preservation analysis of the cDC2 modules in cDC1. The module's size versus the preservation statistic (Z preservation) is shown for each module. $Z < 5$: not preserved; $10 > Z \geq 5$: moderately preserved; $Z \geq 10$: highly preserved **(E)** Module preservation analysis of the cDC2 modules in pDC. **(F)** Enriched GO terms of genes in red module. **(G)** Projection of cDC2 B-related red module in cDC1 subtypes. **(H)** The expression level of projected cDC2 gene modules in cDC1 across different groups. **(I)** Projection of cDC2 B-related red module in pDC subtypes. **(J)** The expression level of projected cDC2 gene modules in pDC across different groups.

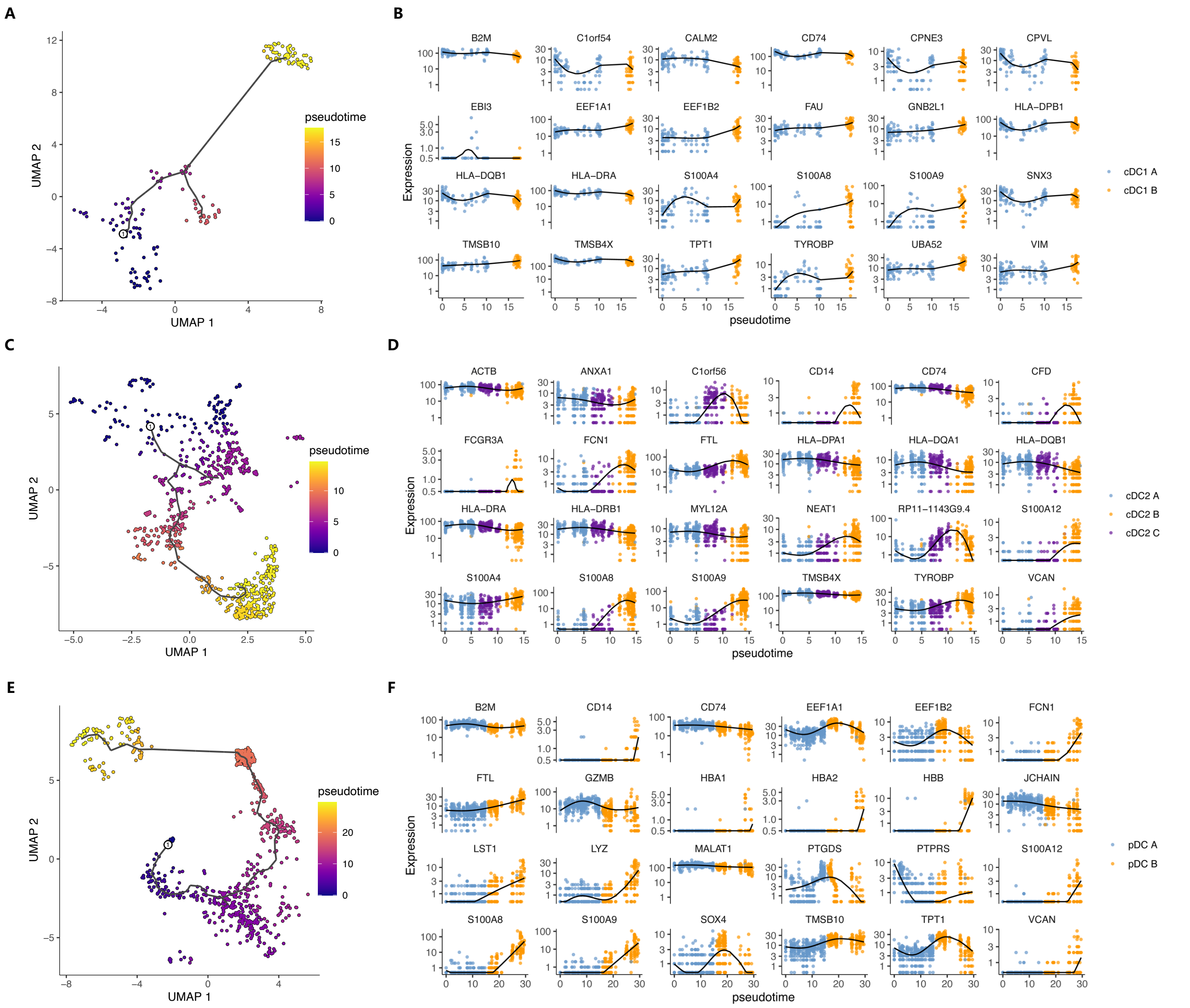

Figure S3. Trajectory and pseudotime analysis of cDC1, cDC2 and pDC in sepsis. **(A)** UMAP embedding of cDC1 colored by inferred pseudotime. **(B)** Scatter plots for the expression patterns of the top differentially expressed genes along the pseudotime of cDC1 subtypes. **(C)** UMAP embedding of cDC2 colored by inferred pseudotime. **(D)** Scatter plots for the expression patterns of the top differentially expressed genes along the pseudotime of cDC2 subtypes. **(E)** UMAP embedding of pDC colored by inferred pseudotime. **(F)** Scatter plots for the expression patterns of the top differentially expressed genes along the pseudotime of pDC subtypes.

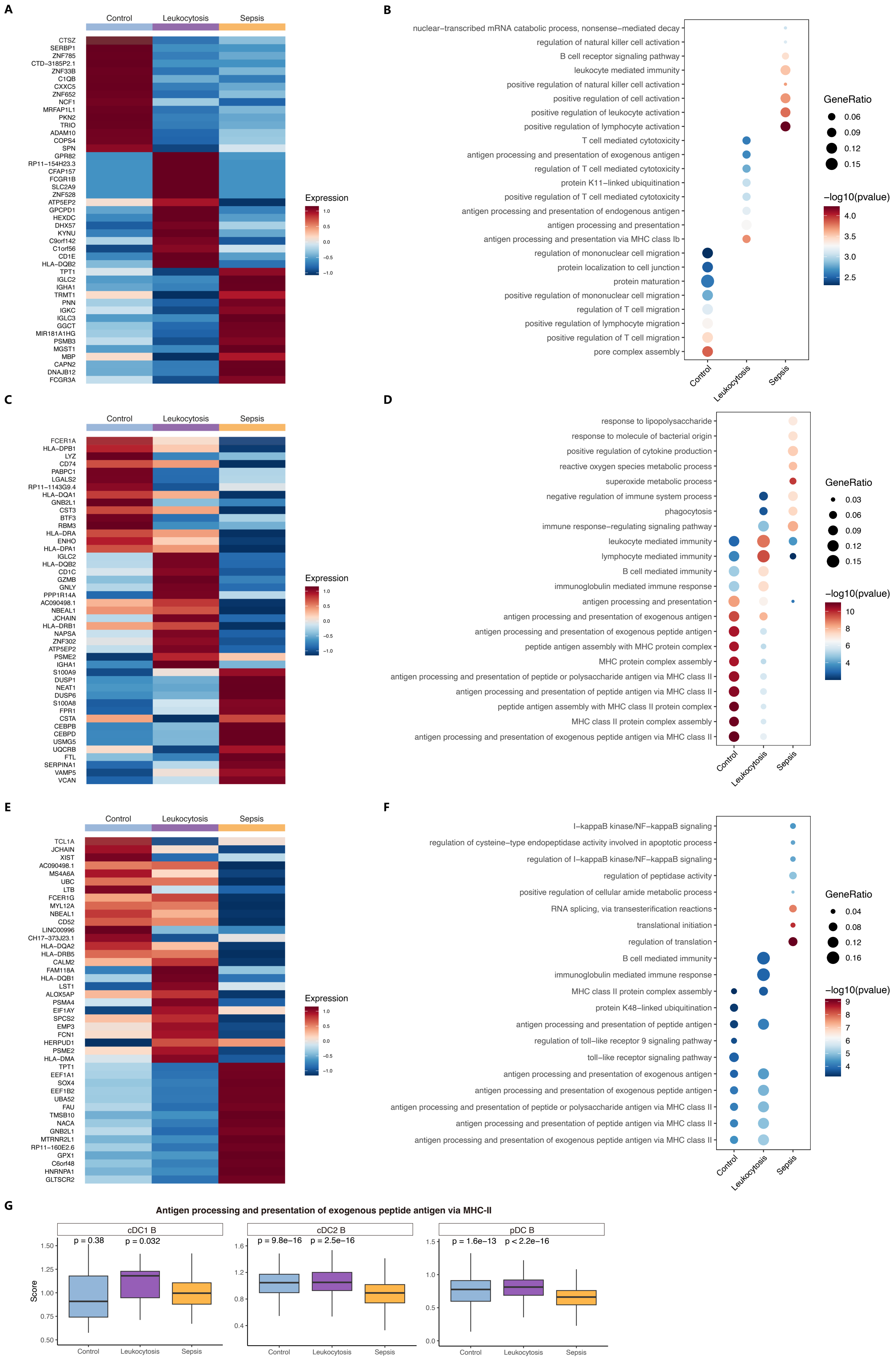
A
Control
Leukocytosis
Sepsis
CTSZ
SERBP1
ZNF785
CTD−3185P2.1
ZNF33B
C1QB
CXXC5
ZNF652
NCF1
MRFAP1L1
PKN2
TRIO
ADAM10
COPS4
SPN
GPR82
RP11−154H23.3
CFAP157
FCGR1B
SLC2A9
ZNF528
ATP5EP2
GPCPD1
HEXDC
DHX57
KYNU
C9orf142
C1orf56
CD1E
HLA−DQB2
TPT1
IGLC2
IGHA1
TRMT1
PNN
IGKC
IGLC3
GGCT
MIR181A1HG
PSMB3
MGST1
MBP
CAPN2
DNAJB12
FCGR3A
Expression
1.0
0.5
0.0
−0.5
−1.0
B
nuclear−transcribed mRNA catabolic process, nonsense−mediated decay
regulation of natural killer cell activation
B cell receptor signaling pathway
leukocyte mediated immunity
positive regulation of natural killer cell activation
positive regulation of cell activation
positive regulation of leukocyte activation
positive regulation of lymphocyte activation
T cell mediated cytotoxicity
antigen processing and presentation of exogenous antigen
regulation of T cell mediated cytotoxicity
protein K11−linked ubiquitination
positive regulation of T cell mediated cytotoxicity
antigen processing and presentation of endogenous antigen
antigen processing and presentation
antigen processing and presentation via MHC class Ib
regulation of mononuclear cell migration
protein localization to cell junction
protein maturation
positive regulation of mononuclear cell migration
regulation of T cell migration
positive regulation of lymphocyte migration
positive regulation of T cell migration
pore complex assembly
Control
Leukocytosis
Sepsis
GeneRatio
0.06
0.09
0.12
0.15
−log10(pvalue)
4.0
3.5
3.0
2.5
C
Control
Leukocytosis
Sepsis
FCER1A
HLA−DPB1
LYZ
CD74
PABPC1
LGALS2
RP11−1143G9.4
HLA−DQA1
GNB2L1
CST3
BTF3
RBM3
HLA−DRA
ENHO
HLA−DPA1
IGLC2
HLA−DQB2
CD1C
GZMB
GNLY
PPP1R14A
AC090498.1
NBEAL1
JCHAIN
HLA−DRB1
NAPSA
ZNF302
ATP5EP2
PSME2
IGHA1
S100A9
DUSP1
NEAT1
DUSP6
S100A8
FPR1
CSTA
CEBPB
CEBPD
USMG5
UQCRB
FTL
SERPINA1
VAMP5
VCAN
Expression
1.0
0.5
0.0
−0.5
−1.0
D
response to lipopolysaccharide
response to molecule of bacterial origin
positive regulation of cytokine production
reactive oxygen species metabolic process
superoxide metabolic process
negative regulation of immune system process
phagocytosis
immune response−regulating signaling pathway
leukocyte mediated immunity
lymphocyte mediated immunity
B cell mediated immunity
immunoglobulin mediated immune response
antigen processing and presentation
antigen processing and presentation of exogenous antigen
antigen processing and presentation of exogenous peptide antigen
peptide antigen assembly with MHC protein complex
MHC protein complex assembly
antigen processing and presentation of peptide or polysaccharide antigen via MHC class II
antigen processing and presentation of peptide antigen via MHC class II
peptide antigen assembly with MHC class II protein complex
MHC class II protein complex assembly
antigen processing and presentation of exogenous peptide antigen via MHC class II
Control
Leukocytosis
Sepsis
GeneRatio
0.03
0.06
0.09
0.12
0.15
−log10(pvalue)
10
8
6
4
E
Control
Leukocytosis
Sepsis
TCL1A
JCHAIN
XIST
AC090498.1
MS4A6A
UBC
LTB
FCER1G
MYL12A
NBEAL1
CD52
LINC00996
CH17−373J23.1
HLA−DQA2
HLA−DRB5
CALM2
FAM118A
HLA−DQB1
LST1
ALOX5AP
PSMA4
EIF1AY
SPCS2
EMP3
FCN1
HERPUD1
PSME2
HLA−DMA
TPT1
EEF1A1
SOX4
EEF1B2
UBA52
FAU
TMSB10
NACA
GNB2L1
MTRNR2L1
RP11−160E2.6
GPX1
C6orf48
HNRNPA1
GLTSCR2
Expression
1.0
0.5
0.0
−0.5
−1.0
F
I−kappaB kinase/NF−kappaB signaling
regulation of cysteine−type endopeptidase activity involved in apoptotic process
regulation of I−kappaB kinase/NF−kappaB signaling
regulation of peptidase activity
positive regulation of cellular amide metabolic process
RNA splicing, via transesterification reactions
translational initiation
regulation of translation
B cell mediated immunity
immunoglobulin mediated immune response
MHC class II protein complex assembly
protein K48−linked ubiquitination
antigen processing and presentation of peptide antigen
regulation of toll−like receptor 9 signaling pathway
toll−like receptor signaling pathway
antigen processing and presentation of exogenous antigen
antigen processing and presentation of exogenous peptide antigen
antigen processing and presentation of peptide or polysaccharide antigen via MHC class II
antigen processing and presentation of peptide antigen via MHC class II
antigen processing and presentation of exogenous peptide antigen via MHC class II
Control
Leukocytosis
Sepsis
GeneRatio
0.04
0.08
0.12
0.16
−log10(pvalue)
9
8
7
6
5
4
G
Antigen processing and presentation of exogenous peptide antigen via MHC-II
cDC1 B
p = 0.38
p = 0.032
Score
1.50
1.25
1.00
0.75
0.50
Control
Leukocytosis
Sepsis
cDC2 B
p = 9.8e−16
p = 2.5e−16
1.6
1.2
0.8
0.4
Control
Leukocytosis
Sepsis
pDC B
p = 1.6e−13
p < 2.2e−16
1.5
1.0
0.5
0.0
Control
Leukocytosis
Sepsis

Figure S4. Phenotypic differences of anergic DCs in septic patients and controls. **(A)** Top upregulated marker genes of cDC1 B in three groups. **(B)** Dot plot showing top enriched pathways of cDC1 B in three groups. Pathways in Gene Ontology biological process database were used. **(C)** Top upregulated marker genes of cDC2 B in three groups. **(D)** Dot plot showing top enriched pathways of cDC2 B in three groups. **(E)** Top upregulated marker genes of pDC B in three groups. **(F)** Dot plot showing top enriched pathways of pDC B in three groups. **(G)** Boxplot showing the antigen-presenting capacity via MHC-II pathway in anergic DCs across septic patients and controls. Significance was determined using Wilcoxon signed-rank test.

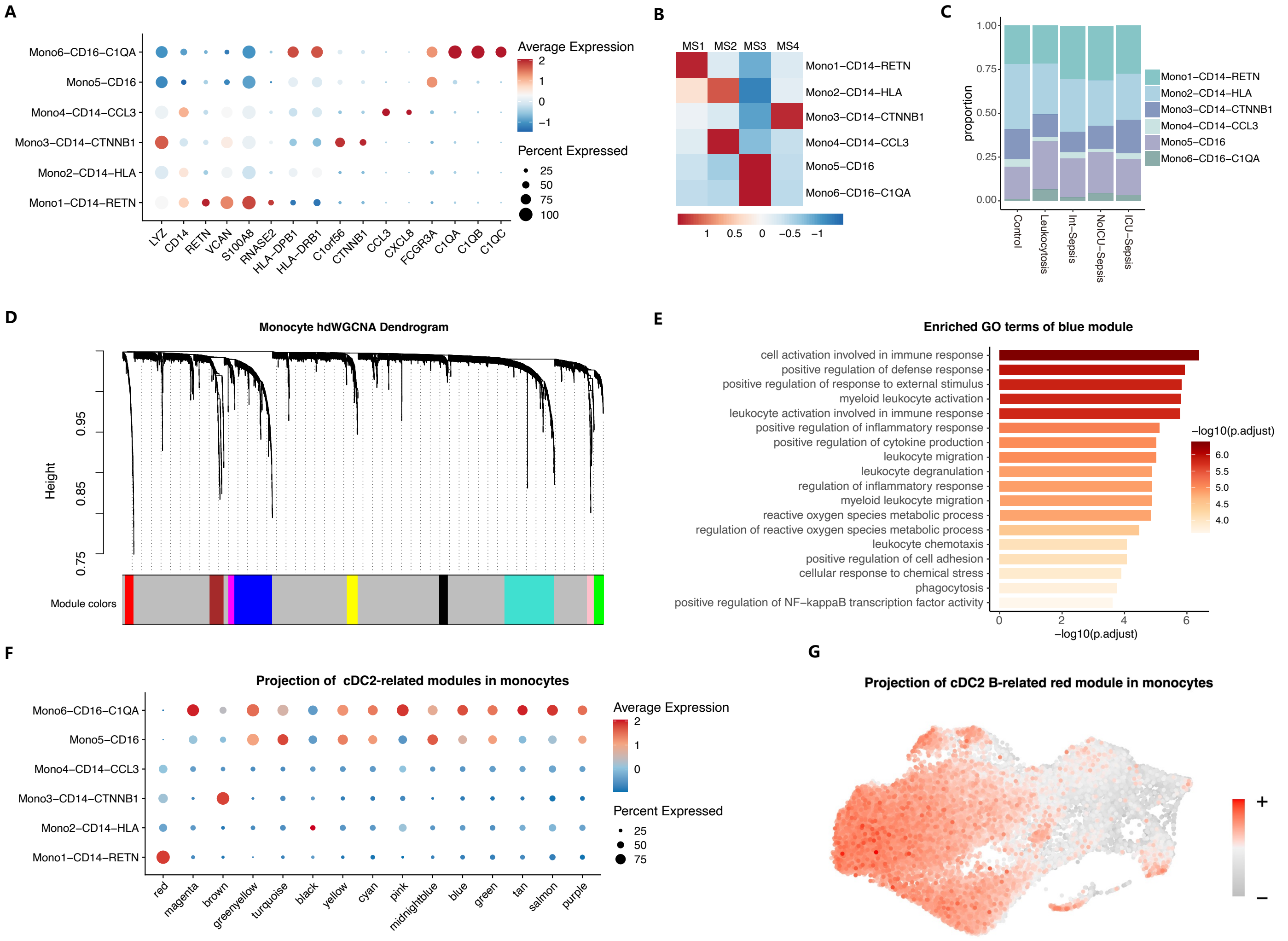

Figure S5. Characteristics of different monocyte subtypes. **(A)** Dot plot for marker genes of six monocyte subtypes. **(B)** Heatmap illustrating the normalized frequency of overlapping cell-type annotations between Reyes et al's findings and those from this study. The data were scaled by row. **(C)** Bar plot showed six monocyte subtypes' proportion in different groups**. (D)** The cluster dendrogram of co-expression genes of monocyte. **(E)** Enriched GO terms of genes in Mono1-related blue module. **(F)** The expression level of projected cDC2 gene modules in Mono1 across different groups. **(G)** Projection of cDC2-B specific red module in monocyte subtypes.

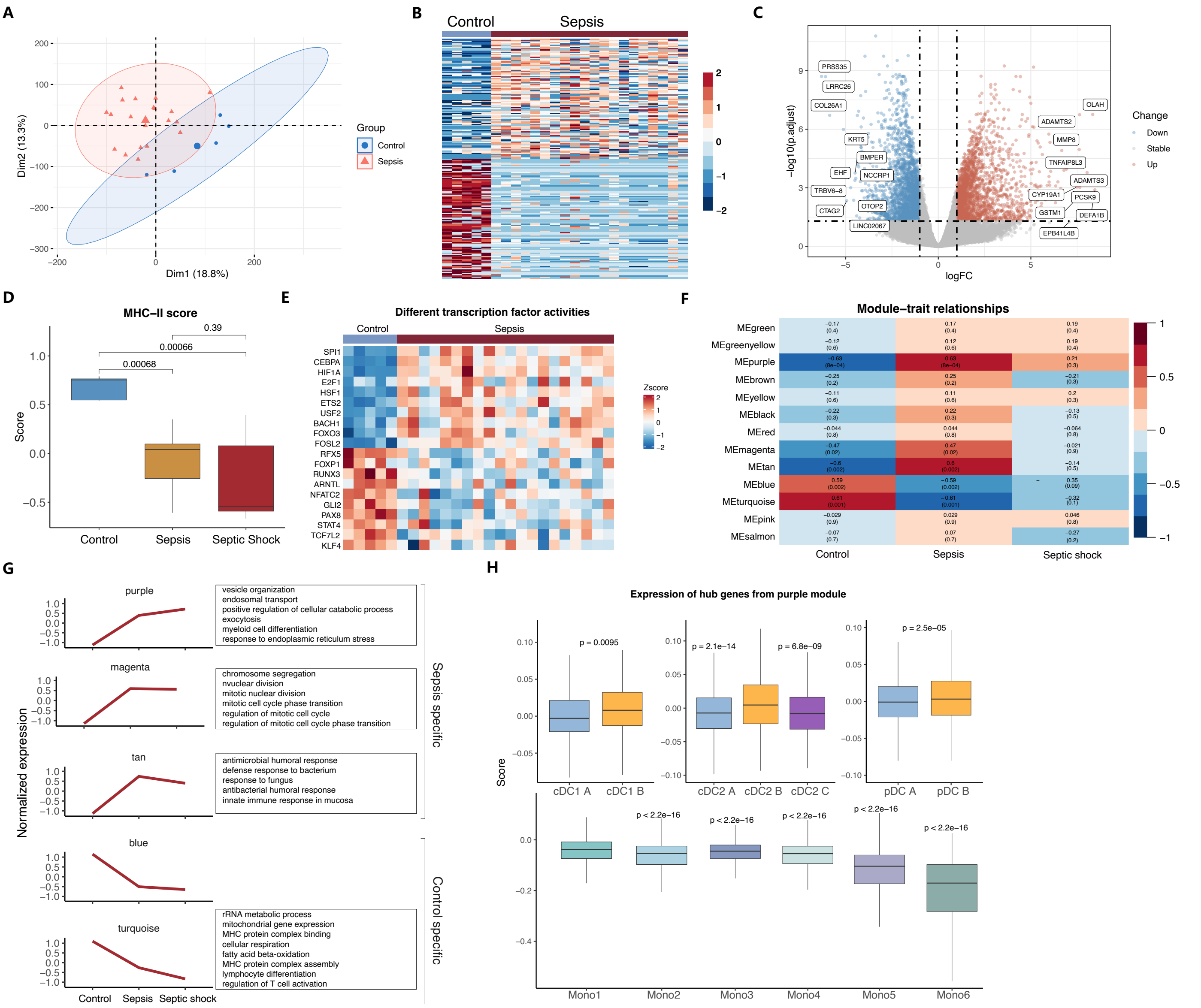

Figure S6. Different characteristics between septic patients and healthy controls. **(A)** Principal component analysis of healthy controls and septic patients. **(B)** Heatmap of top DEGs in sepsis versus control. **(C)** Volcano plot of differentially expressed genes between septic patients and healthy controls. **(D)** Gene-set score of MHC-II related genes between control, sepsis and septic shock groups. Significance was determined using Student's t test. **(E)** Heatmap showed top different expressed TFs. **(F)** Heatmap representation of module-trait relationships. Each cell contains the related correlation and P-value. **(G)** Line chart showing the different expression levels of 5 co-expression modules in control, sepsis and septic shock groups (left). Enriched GO terms of each module (right). **(H)** Expression of hub genes from purple module identified in bulk RNA-seq data across DC subtypes (top) and monocyte subtypes (bottom). Hub genes with

|GS|>0.7 and |MM|>0.8 were included in the analysis. Significance was determined using Wilcoxon signed-rank test.
